# Snowflake morays, *Echidna nebulosa*, exhibit similar feeding kinematics in terrestrial and aquatic treatments

**DOI:** 10.1242/jeb.234047

**Published:** 2021-06-10

**Authors:** Rita S. Mehta, Kyle R. Donohoe

**Affiliations:** Department of Ecology and Evolutionary Biology, University of California, Santa Cruz, CA 95064, USA

**Keywords:** Pharyngeal transport, Muraenidae, Elongate body plan

## Abstract

Some species of durophagous moray eels (Muraenidae) have been documented emerging from the marine environment to capture intertidal crabs but how they consume prey out of water is unknown. Here, we trained snowflake morays, *Echidna nebulosa*, to undulate out of the aquatic environment to feed on land. On land, snowflake morays remove prey from the substrate by biting and swallow prey using pharyngeal jaw enabled transport. Although snowflake morays exhibit smaller jaw rotation angles on land when apprehending their prey, transport kinematics involving dorsoventral flexion of the head to protract the pharyngeal jaws and overall feeding times did not differ between terrestrial and aquatic treatments. We suggest that their elongate body plan, ability to rotate their heads in the dorsoventral and lateral directions, and extreme pharyngeal movements all contribute to the ability of durophagous morays to feed in the terrestrial environment.

## Abstract (precis)

**Summary:** Body elongation and pharyngeal transport facilitates prey capture and swallowing on land for the snowflake moray, *Echidna nebulosa*.

## INTRODUCTION

Many extant fish taxa are able to make terrestrial excursions (Johnels, 1957; Chave and Randall, 1971; Pace and Gibb, 2011, 2014; Kawano and Blob, 2013; Standen et al., 2016; Bressman et al., 2019) and either breathe air or withstand hypoxia for extended periods of time (Sayer, 2005). Studies focused on amphibious species have mainly examined the kinematics of fishes moving on land (Hsieh, 2010; Pace and Gibb, 2011; Kawano and Blob, 2013; Ward et al., 2015; Mehta et al., 2020), physiological adaptations to the terrestrial environment (LeBlanc et al., 2010; Turko et al., 2019) and the genetics therein (You et al., 2018). Fewer studies have explored other behaviors while on land such as socialization (Taylor et al., 2008; Ord and Hsieh, 2011) and feeding (Seghers, 1978; Van Wassenbergh et al., 2006; Van Wassenbergh, 2013; Michel et al., 2014, 2015a,b). Feeding and seeking novel resources are especially interesting hypotheses for why vertebrates may have invaded the terrestrial environment around 400 MYA (Long and Gordon, 2004; Ashley-Ross et al., 2013). Extant fishes that have been documented moving onto land to take advantage of novel food resources tend to rely on the aquatic environment to manipulate and swallow prey (Cucherousset et al., 2012) or are able to hold water in their buccal cavity while on land to use it for feeding (Michel et al., 2015b). The need for water is not surprising as the vast majority of fishes rely on hydraulic transport to move prey from the buccal cavity into the esophagus (Lauder, 1983).

Muraenid fishes (Anguilliformes) collectively known as morays are good models for examining the functional morphology of feeding in aquatic and terrestrial environments. Morays apprehend their prey by biting and use pharyngeal transport to move prey from their buccal cavity into their esophagus (Mehta and Wainwright, 2007a,b). Pharyngeal transport in muraenids does not rely on water movement, suggesting that morays should be able to capture and swallow prey on land, although transport is not the only challenge fishes feeding on land must overcome (see review in Heiss et al., 2018). Morays also tend to be relatively robust to hypoxic conditions (Ackerman and Bellwood, 2000) and a histological study on the skin of the Mediterranean moray, *Muraena helena*, revealed phospholipids and unicellular mucous glands in the outermost layer of the epidermis which may delay desiccation (Zaccone, 1979). Observations from the field also report that a few species of morays, *Gymnothorax pictus* (Chave and Randall, 1971), *Echidna nebulosa* (Fishelson, 1977) and *Sidera grisea* (Fishelson, 1977; R.S.M., unpublished observations) move into or even above the intertidal zone to feed on crabs. Most recently, *G. pictus* have been described ingesting their prey, whole shore crabs, *Graspus tenicrustatus*, while remaining partially or fully emerged from the water (Graham et al., 2009). However, the mechanics underlying feeding while a moray is partially or fully emerged from the water have not been described. While the kinematics of aquatic prey capture in the snowflake moray, *E. nebulosa*, has been quantified (Mehta and Wainwright, 2007a), the goal of this study was to extend the analysis of feeding kinematics to the terrestrial environment. We hypothesized that feeding in two physically different environments would require differences in skull kinematics for snowflake morays. We predicted that skull movements within the two phases of the feeding cycle, prey apprehension and prey transport, would be similar in magnitude but that feeding durations between terrestrial and aquatic treatments would differ.

## MATERIALS AND METHODS

Seven individuals of the muraenid eel, *Echidna nebulosa* (Ahl 1789), were obtained commercially and maintained in separate ∼76 liter aquaria. Size of individuals are reported in Table S1. The age and sex of individuals were unknown. Each aquarium had a fluval canister filter system, an aerator, a thermometer and 1–2 sections (150 mm long) of PVC pipe as a retreat. Temperature and salinity were maintained between 24 and 26°C and 33–35 ppm. Individuals were fed a diet of cut up squid (*Loligo* sp.) or octopus (*Octopus vulgaris*) to satiation weekly.

To determine whether feeding behavior varies between terrestrial and aquatic environments, we built Plexiglas ramps ∼3 mm thick and 25×18 mm, and angled at 25 deg. These ramps led to 12×12 mm Plexiglas platforms. The platform and ramp were glued into one side of the individual's home tank with all-glass clear silicone aquarium sealant. The ramp was lightly coated in epoxy resin and sprinkled with sand to reduce slip. The design was such that eels had to learn to associate food with the ramp or platform. The platform and upper half of the ramp were above the water line, and from herein are designated as the terrestrial treatment.

Training and video recordings for different snowflake moray individuals took place over a 6 year period by a variety of student trainers. No single moray was involved in this experiment for more than 8 months. All training took place in an individual's home tank to minimize stress. Through the process of positive reinforcement and shaping (rewarding the individual for incremental progress towards the desired behavior), fish learned to feed on the ramp or platform without retreating to the water.

During shaping, prey was presented ∼2.5–5 cm between the ramp/water interface. The water line was approximately half-way up the ramp. Once the eel's head emerged from the water and grabbed onto the prey, we held onto the prey with forceps and pulled the eel further onto the ramp where more prey was located. This process, where the subject is physically manipulated to the desired behavior or position, is termed molding. After successive weeks of feeding, if the eel was hauling out onto the ramp without the aid of a trainer, we lowered the water level to 1/3rd the length of the ramp. At this lowered water level, when the eel hauled out onto the ramp and apprehended prey, we again maintained our hold on the prey with forceps and gradually pulled the eel up to the platform. We started video recording individual snowflake morays feeding when both prey apprehension and transport took place on the ramp and there was no interference by the researcher, so that eels learned to both undulate out of water and feed in the terrestrial environment on their own. In order to compare feeding parameters between terrestrial and aquatic treatments, morays were required to stay in one medium during the entirety of the feeding event (prey apprehension and transport). These video recordings ranged from morays being lured up onto the ramp with food and then pulled further up the ramp once they grabbed onto the prey with their oral jaws, to the trainer leaving pieces of prey on the ramp without further interacting with the eel. When eels were pulled further up onto the ramp by the trainer, feeding behavior was not analyzed unless the eel remained on the ramp and continued to capture other pieces of prey unassisted. For aquatic treatments, pieces of prey were offered with forceps in water and released once the eel approached the prey; prey apprehension and transport were entirely submerged.

Feeding trials for both terrestrial and aquatic treatments were recorded with a Panasonic Lumix DMC-FZ1000 camera. Individuals were offered squid pieces that were 3 mm by 30 mm. These prey dimensions were ideal for this study because they were small enough that feeding on a single prey item would not result in satiation, which could influence feeding kinematics (Sass and Motta, 2002). Also, offering prey of a rectangular shape rather than a square shape provided length that elicited head movements, which enabled us to observe multiple protraction and retraction cycles during transport. Our largest individual was offered pieces of squid that were much wider but were the same length. Motivation drove the discrepancy in prey size for this individual as smaller pieces of squid would not encourage terrestrial feeding. Feeding trials were taken at 120 frames s^−1^ and used 1–4 pieces of squid. Feeding trials between the two treatments (terrestrial versus aquatic) were conducted separately. Animals were fed uniform pieces of squid one after another in both treatments but there was considerable variation in how long morays would take to undulate their bodies out from the water as a result of individual animal motivation and variation in training conditions. Table S1 provides the number of videos obtained in each of the treatments experienced, the number of kinematic acquisition and transport (a, t) events quantified within each treatment, and various size dimensions of the individual morays. All husbandry and experiments were approved by IACUC protocol (IACUC #1009).

### Analysis

Feeding trials where at least part of the feeding cycle was in lateral view were saved digitally and played back for analysis using the free video analysis software Tracker (https://physlets.org/tracker/). We measured the following kinematic variables: jaw rotation angle (deg), calculated from the largest gape distance between the upper and lower jaws during the prey apprehension phase just prior to when the jaws make contact with prey; dorsoventral flexion (deg), the angle at which the eel's head dorsoventrally flexes to protract the pharyngeal jaws at the beginning of the prey transport phase; the number of protraction–retraction cycles – this behavior was distinct as it began with dorsoventral flexion of the head (protraction) to elevation of the head (retraction) during prey transport; and total feeding time (s), defined as the time from when the eel made contact with prey with its oral jaws to the last protraction–retraction behavior during prey transport and the prey was no longer visible. As morays consumed up to four prey items within each feeding trial, we calculated averages for each of the kinematic variables across feeding events within a single trial and performed the analyses on the kinematic averages for each individual.

To determine whether size influenced kinematics variables, we regressed head size against the four kinematic variables. Head size was calculated using the geometric mean of head length (mm), height (mm) and width (mm). We used head size rather than standard length because all kinematic variables we quantified were associated with the head. None of the kinematic variables showed a relationship with head size. We then log-transformed total feeding time to reduce skew and normalize the variance for statistical analyses. We tested for equal variance using a Levene's test. We then used a one-way multivariate analysis of variance (MANOVA) to determine whether our dependent variables, jaw rotation angle, dorsoventral flexion, number of protraction–retraction cycles and total feeding time, differed between treatment (terrestrial versus aquatic), which was the main effect. We therefore linked prey apprehension and transport behaviors in the single model. Analyses were performed using the function MANOVA in car package of R software (http://www.R-project.org/).

## RESULTS AND DISCUSSION

We analyzed a minimum of 3 feeding trials for each individual in the terrestrial treatment (range 3–9) and a minimum of 2 feeding trials for aquatic treatments (range 2–10). The majority of individuals learned to feed on the ramp, and many searched for prey at the water–ramp interface without coaxing from a trainer (Movie 1). Two individuals were observed voluntarily undulating up the ramp and then onto the platform before food was presented, but this behavior was inconsistent. For the majority of terrestrial trials, snowflake morays undulated the upper 1/3rd of their body from the water to capture prey on the ramp.

All seven snowflake morays were recorded feeding in the terrestrial treatment (Table S2); only five individuals were recorded feeding in both terrestrial and aquatic treatments. Our statistical analyses, used to determine whether kinematic differences existed between feeding in the terrestrial and aquatic treatments, incorporated four of the five individuals that experienced both treatments. The larger piece of squid that was used to motivate the fifth and largest individual to feed in both environments confounded our dataset. Therefore, while data on the kinematic averages for this individual are presented in Table S2, they were not included in our statistical analyses.

**Fig. 1.**
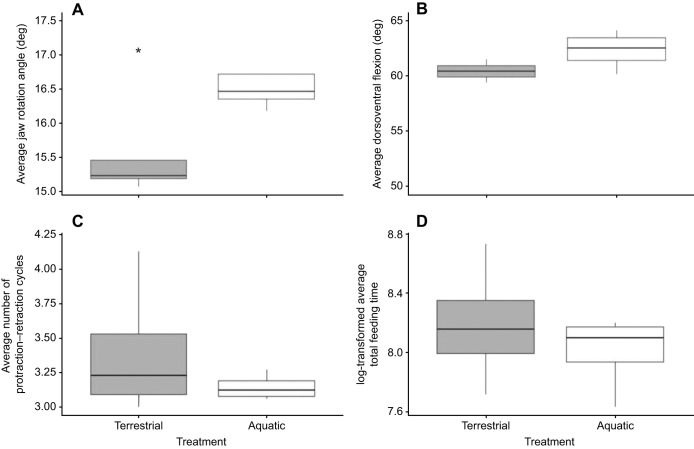
**Box plots showing the effects of treatment (terrestrial/aquatic) on each of the four kinematic variables.** (A) Average jaw rotation angle, (B) average dorsoventral flexion, (C) average number of protraction–retraction cycles, and (D) log-transformed average total feeding time. Average jaw rotation angle was the only kinematic variable that significantly differed between treatments (*P=0.014).

Of the 85 feeding trials recorded, 67 videos provided lateral views of the feeding sequence that were long enough to quantify combinations of three of the four kinematic variables contributing to prey acquisition and transport. Means for jaw rotation, dorsoventral flexion angle of the head, and the number of protraction–retraction behaviors for each treatment are reported in Table S2. The MANOVA revealed that treatment (terrestrial or aquatic) had an effect on only one kinematic parameter, jaw rotation angle (*F*_1,6_=11.79, *P*=0.014), which was significantly smaller in the terrestrial treatment (Fig. 1A).

The kinematic variables associated with the transport phase of feeding, dorsoventral flexion (*F*_3,6_=1.65, *P*=0.28; Fig. 1B) and protraction–retraction behavior (*F*_3,6_=5.75, *P*=0.34; Fig. 1C) did not differ between treatments. Lastly, there were no differences in average total feeding time between terrestrial and aquatic treatments (*F*_1,6_=0.538, *P*=0.49; Fig. 1D).

Our study shows that snowflake morays can apprehend and swallow prey out of water. Interestingly, we found little difference in kinematic variables for feeding between terrestrial and aquatic environments. Although many extant species of fish are able to make terrestrial excursions, only the mudskipper has been shown to use a hydrodynamic tongue and pharyngeal jaws for swallowing prey in the terrestrial environment (Sponder and Lauder, 1981; Michel et al., 2015b). Unlike mudskippers, snowflake morays are not dependent on water to move prey further into the mouth cavity as their pharyngeal jaws have considerable protraction distances compared with those of other fishes and can transport prey from their oral jaws into their esophagus. It has been hypothesized that the extreme protraction of the pharyngeal jaws into the oral cavity enables moray eels to maintain at least one set of jaws on struggling prey in the marine environment (Mehta and Wainwright, 2007a,b). While there is a paucity of data on moray behaviors in the wild, the published accounts of morays moving on land to hunt for prey are for durophagous morays, those consuming prey encased in a shell such as crabs. Our data suggest that pharyngeal transport also facilitates the ability of morays to swallow prey while on land (Movie 1).

We had a relatively narrow size range of morays that we could include in our statistical analyses (standard length 233–314 mm, head length 17–28 mm). Our largest individual (standard length 520 mm, head length 42.5 mm), which was omitted from statistical analyses because of confounds with prey size, exhibited a lower number of protraction–retraction events and shorter mean total feeding times (Movie 2). Unfortunately, whether these fewer and quicker movement patterns were due to moray size or the fact that the prey offered were not sufficiently scaled to the size of the moray is not known. The published accounts of morays feeding on crabs in the intertidal or above the intertidal line do not report the size of the morays observed. Observations of *Siderea griseus* moving onto land to search for crabs on a protected island in South Sulawesi, Indonesia, were for individuals that were roughly 600 mm in total length (R.S.M., unpublished observations), which is close to the maximum size reported for the species. In the wild, whether larger individuals are more likely than smaller individuals to make forays into the intertidal zone or actively emerge from the water and successfully feed on land is an interesting question for future investigation.

Researchers have recognized six mechanical problems that a suction-feeding fish would need to circumvent in order to capture and transport prey on land (Heiss et al., 2018). The majority of these challenges (i.e. stabilizing the body on land and relaxation of mechanical constraints on head movements to effectively apprehend prey) are overcome by highly elongate fishes lacking or with reduced pectoral fins. Eel-like fishes such as the reedfish (*Erpetocihthys calabaricus*) and the eel catfish (*Channallabes apus*) are cylindrical rather than laterally compressed in body shape. This cylindrical body shape allows them to maintain stable postures on land and throughout the prey capture event. During prey capture, both fishes lift a portion of their trunk up off the ground and dorsoventrally rotate their head to aim their gape downwards at the prey, which also allows the hyoid to move freely and avoid contact with the ground. Snowflake morays are also able to orient their gape downwards by having a pseudo ‘neck’ facilitated by their elongate body plan. Biting is also the dominant mode of prey capture so the reedfish and eel catfish are not reliant on water during this capture stage. However, these species are unable to circumvent the mechanical problem of swallowing without water as they must either (1) retreat back to the water with the prey to use hydrodynamic transport or as in the case of mudskippers (2) maintain water in their buccal cavity (Heiss et al., 2018, and references therein).

**Fig. 2.**
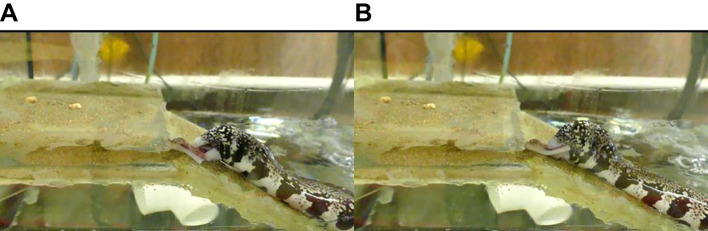
**Representative images of a snowflake moray feeding on the ramp in the terrestrial treatment.** (A) The moray rotates its head laterally to bite the prey after attempts to bite the prey by dorsoventral flexion of the head were not successful. (B) The moray uses dorsoventral flexion of the skull, marking the beginning of prey transport.

In our study, snowflake morays were able to circumvent the mechanical problems recognized by Heiss et al. (2018) and use a more extreme pharyngeal transport than what has been reported for the amphibious mudskippers (Sponder and Lauder, 1981). Thus, the ability of snowflake morays to feed on land is partly attributed to their elongate body plan and their highly mobile pharyngeal jaw system. The shorter heads of durophagous morays (Collar et al., 2014; Gartner and Mehta, 2020) and their elongate bodies enable them to move their heads in the dorsoventral direction and also laterally rotate their heads (Fig. 2A). While the flat pieces of squid prey were consistent in size for the majority of morays, they were challenging for morays to bite with their oral jaws. Prey capture often involved morays moving their heads dorsoventrally and using multiple attempts to open and close their mouths around the prey. In 67% of terrestrial trials, individuals exhibited lateral rotation of the skull to effectively orient their jaws around the prey when dorsoventrally oriented biting was unsuccessful (Fig. 2A; Movie 3). In this lateral orientation of prey capture, quantifying gape distance was not possible. Once prey was grasped, the moray would reorient its head and then dorsoventrally flex the skull to begin the transport phase of feeding (Fig. 2B). Durophagy in morays is associated with morphological features such as a shorter head, higher mechanical advantage of the lower jaw, larger adductor mandibulae muscles and short, blunt or molariform dentition (Collar et al., 2014; Mehta, 2009). The short oral jaws of durophagous morays paired with head mobility may be beneficial for feeding terrestrially. Many elongate fishes have increased skull mobility to varying degrees as a result of the propensity for elongate fishes to reduce or lose their pectoral fin connection with the skull (Ward and Mehta, 2010). Piscivorous morays may learn to locate amphibious fishes occupying the intertidal, isolated shallow pools, or mangal pools (Bennett, 2010) but whether their longer oral jaws facilitate biting prey on land and acquiring prey by moving their head dorsoventrally and laterally necessitates investigation.

We successfully trained snowflake morays to feed out of water. The only individual that we still have in the lab contributed to the study in 2014. This individual has retained the behavior of feeding on the ramp and will often undulate onto the ramp before prey is even presented. This individual voluntarily feeds on the ramp for as many as four pieces of prey at a time, without needing to return to the water in between each feeding event. Creating an association between prey and a novel environment (the ramp) enabled us to ‘teach’ these animals to feed in a new context. Learned behaviors in fishes are largely unstudied, and this study provides an example of long-term retention of a feeding task.

Experimental studies that build on observation from individuals in the wild are valuable as they provide useful insights into how certain fishes may navigate different environments and take advantage of available resources. We can discover how some species may be resilient during challenging times or opportunistic during the cycle of tides. While many studies of amphibious fishes have helped to advance our understanding of the aquatic–terrestrial feeding transitions in vertebrate evolution, it is unclear whether pharyngeal transport in morays provides us with insight into fishapod feeding or the first transitional tetrapods. For many extant fish species, the pharyngeal jaws play an important role packing prey into the esophagus (Vandewalle et al., 1994). However, the extreme mobility of the pharyngeal jaws is highly specialized in moray eels and it is unknown whether Devonian fishes had any extreme pharyngeal movement. We do show that a durophagous moray eel, such as the snowflake moray, that exhibits a relatively broad geographic distribution, has the ability to affect multiple ecosystems by consuming fully aquatic, amphibious and terrestrial prey.

## Supplementary Material

Supplementary information
