# Supplementary material for: Snowflake morays, Echidna nebulosa, exhibit similar feeding kinematics in terrestrial and aquatic treatments
Source: J Exp Biol. 2021 Jun 10;224(11):jeb234047. doi: 10.1242/jeb.234047 (PMC8214832; doi:10.1242/jeb.234047)
Supplement: Supplementary information [file jexbio-224-234047-s1.pdf]

Table S1. Number of videos obtained in each of the treatments experienced, number of kinematic acquisition and transport (a, t) events quantified within each treatment, and various size dimensions of the snowflake morays (*Echidna nebulosa*, n=7) that participated in the feeding trials from June 2014 to May 2020. *Indicates individuals that were not included in statistical analyses because prey size was not comparable or because they only fed in the terrestrial treatment.

	Treatments Experienced		Size Measurements (mm)			
**Individual**	**Terrestrial Total (a,t)**	**Aquatic Total (a,t)**	**Head Length**	**Head Height**	**Head Width**	**Standard Length**
Benjen*	9 (6, 4)	4 (4,4)	42.5	24	22	520
Marsh	3 (3,3)	2 (2,2)	17	9	7.75	233
Qani	4 (4,4)	4 (4,4)	20.5	12	9	262
Jetsom	6 (4,5)	10 (8, 9)	28	16	11.5	314
Frosty	4 (4,4)	6 (6,6)	17	8.0	8.5	258
Flatsom*	7 (6,5)	0	25	12	9	288
LB*	8 (6,6)	0	27	12	11	296

Table S2. Summary of the four kinematic variables quantified in this study. Means and S.E. for jaw rotation angle, dorsoventral flexion angle of the head, the number of protraction-retraction behaviors, and total feeding time are reported within each treatment.*Individual not included in statistical analyses due to differences in prey size. ** Individuals not included in statistical analyses because they only experienced terrestrial treatments.

	Terrestrial Treatments				Aquatic Treatments			
Individual	Jaw Rotation Angle (degrees)	Dorsoventral Flexion (degrees)	Number of protraction-retractions	Total Feeding Time (s)	Jaw Rotation Angle (degrees)	Dorsoventral Flexion (degrees)	Number of protraction retractions	Total Feeding Time (s)
Benjen*	16.8 ± 0.37	64.3 ± 8.8	2.15 ± 0.6	2.7 ± 1.2	17 ± 0.32	67.6 ± 7.5	1.8 ± .4	1.8 ± .60
Marsh	15.23 ± 0.18	45.3 ± 3.3	3.3 ± 0.6	3.3 ± .50	17.33± 0.32	63.4 ± 3.0	3.25 ± .5	3.9 ± 1.4
Qani	16.13 ± 0.09	59.4 ± 1.7	3.0 ± 0.8	2.3 ± .60	16.52 ± 0.82	61.3 ± 4.5	3.4 ± 1.0	2.5 ± .96
Jetsom	15.24 ± 0.23	65.6 ± 4.1	3.2 ± 0.8	3.8 ± 1.6	16.18 ± 0.73	59.9 ± 4.1	3.0 ± 1.2	3.5 ± 1.9
Frosty	15.07 ± 0.12	61.5 ± 1.8	4.0 ± 1.0	6.4 ± 2.0	16.41 ± 0.21	61.7 ± 6.1	2.9 ± .9	3.1 ± 1.3
Flatsom**	15.32 ± 0.31	56.6 ± 7.2	4.6 ± 1.6	5.5 ± 2.7	NA	NA	NA	NA
LB**	15.3 ± 0.41	69.3 ± 10.4	3.1 ± 1.5	4.9 ± 2.7	NA	NA	NA	NA

Journal of Experimental Biology • Supplementary information

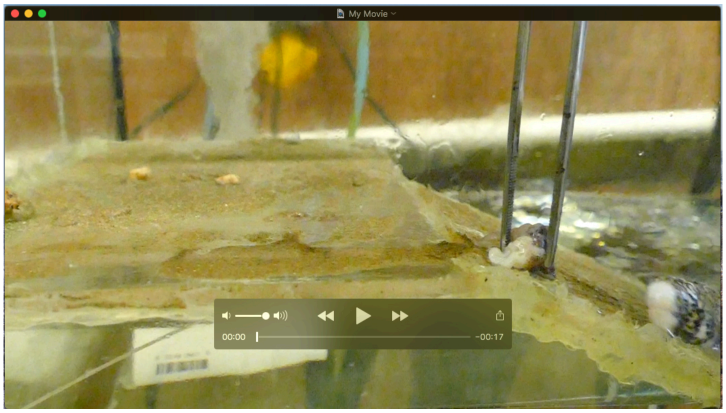

Movie 1. An example of a snowflake moray emerging from the water onto land, apprehending prey with the oral jaws and then transporting prey with its pharyngeal jaws.

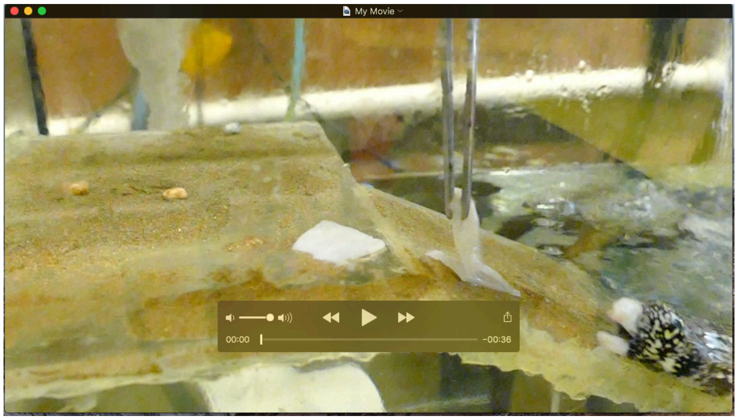

Movie 2. An example of our largest snowflake moray in the dataset consuming prey on the ramp. Notice the dorsoventral flexion of the head during pharyngeal transport.

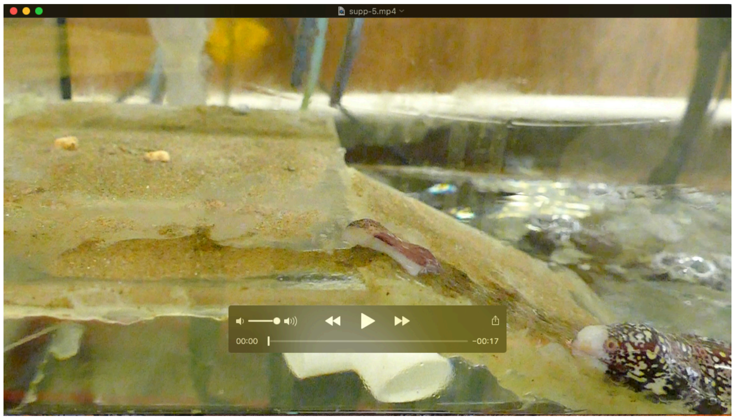

Movie 3. An example of a snowflake moray rotating its head when trying to apprehend flat pieces of prey on the substrate.
